# Supplementary material for: MetaRibo-Seq measures translation in microbiomes
Source: Nat Commun. 2020 Jun 29;11:3268. doi: 10.1038/s41467-020-17081-z (PMC7324362; doi:10.1038/s41467-020-17081-z)
Supplement: Supplementary file 10 — Supplementary Data 7 [file 41467_2020_17081_MOESM10_ESM.zip › File2/Confidence_High_Taxonomy/9281_out.krona.html]

Javascript must be enabled to view this page.

members
magnitude
magnitudeUnassigned
count
unassigned
taxon
rank

9281\_out

3

2
3
superkingdom

phylum
3
1239

909932
1
class

order
1843489
1

31977
1
family

29465
1
genus


SRS147887\_contig\_number\_9844
1
39777
species

1
186801
class

order
186802
1

186803
1
family

genus
1
1164882

979627
1

SRS019022\_contig\_number\_contig-100\_7372.36273
species

class
91061
1

1
186826
order

family
1300
1

1
1301
genus

1305
1

SRS074682\_contig\_number\_contig-100\_924.191542
species
